# Lipid remodeling of adipose tissue in metabolic health and disease

**DOI:** 10.1038/s12276-023-01071-4

**Published:** 2023-09-01

**Authors:** Yoon Keun Cho, Sumin Lee, Jaewon Lee, Junsang Doh, Joo-Hong Park, Young-Suk Jung, Yun-Hee Lee

**Affiliations:** 1 College of Pharmacy and Research Institute of Pharmaceutical Sciences, Seoul National University, Seoul, Republic of Korea; 2 Department of Materials Science and Engineering, Research Institute of Advanced Materials, Institute of Engineering Research, Bio-MAX Institute, Soft Foundry Institute, Seoul National University, Seoul, Republic of Korea; 3 School of Biological Sciences, Seoul National University, Seoul, Republic of Korea; 4 College of Pharmacy, Pusan National University, Busan, Republic of Korea

**Keywords:** Obesity, Metabolomics

## Abstract

Adipose tissue is a dynamic and metabolically active organ that plays a crucial role in energy homeostasis and endocrine function. Recent advancements in lipidomics techniques have enabled the study of the complex lipid composition of adipose tissue and its role in metabolic disorders such as obesity, diabetes, and cardiovascular disease. In addition, adipose tissue lipidomics has emerged as a powerful tool for understanding the molecular mechanisms underlying these disorders and identifying bioactive lipid mediators and potential therapeutic targets. This review aims to summarize recent lipidomics studies that investigated the dynamic remodeling of adipose tissue lipids in response to specific physiological changes, pharmacological interventions, and pathological conditions. We discuss the molecular mechanisms of lipid remodeling in adipose tissue and explore the recent identification of bioactive lipid mediators generated in adipose tissue that regulate adipocytes and systemic metabolism. We propose that manipulating lipid-mediator metabolism could serve as a therapeutic approach for preventing or treating obesity-related metabolic diseases.

## Metabolic disorders: lipid dynamics as biomarkers and therapeutic targets

Large-scale analyses of lipid composition and distribution in body fat (adipose tissue) have the potential to revolutionize the treatment and prevention of metabolic disorders associated with obesity. Yun-Hee Lee from Seoul National University, South Korea, and colleagues review the intricate molecular mechanisms underlying lipid synthesis, breakdown, and redistribution, both in healthy and diseased states. Advances in ‘lipidomics’, the analysis of the complete set of lipids and their reactions, have helped to illuminate the complex interactions between bioactive lipids and lipid-modifying enzymes. Such interactions play a critical roles in insulin sensitivity, energy expenditure, and other metabolic processes. The authors propose that the signaling pathways involved, along with the bioactive lipid mediators themselves, could serve as biomarkers or therapeutic targets for type 2 diabetes, cardiovascular disease, and various obesity-related conditions.

## Introduction

The increasing prevalence of obesity and metabolic disorders has spurred research on the role of adipose tissue in the pathogenesis of metabolic diseases^1^. Adipose tissue is a complex organ involved in the regulation of energy homeostasis and plays a key role in the development of metabolic diseases such as obesity, type 2 diabetes (T2D), and cardiovascular disease (CVD)^1^. Adipose tissue lipid remodeling involves changes in the composition and distribution of lipids in response to environmental and metabolic cues^2^. These changes can have significant effects on adipose tissue function, including alterations in adipocyte differentiation, lipolysis, and insulin sensitivity, and potentially underlie the link between adipose tissue dysfunction and metabolic diseases.

Lipidomics can be defined as a field of study that involves the comprehensive analysis of lipids in biological systems^2^. By identifying specific lipid species and lipid classes that are dysregulated in metabolic diseases, lipidomics studies have shed light on the molecular mechanisms underlying the development and progression of these disorders. These studies have also identified potential biomarkers and therapeutic targets. This review provides an overview of recent lipidomics studies that investigated adipose tissue lipid remodeling and its impact on metabolic health, emphasizing its importance in the development of prevention and treatment strategies for metabolic diseases. This review is organized into three main sections, each examining distinct aspects of lipidomics studies that focus on adipose tissue lipid remodeling. The first section provides an overview of the literature on lipidome remodeling in adipose tissue under various physiological and pathological states. To support the clinical relevance of these findings, we also included lipidomics studies of human plasma, as well as adipose tissue. This inclusion is necessary due to the limited research available on human adipose tissue lipidomics. The second section focuses on the metabolic roles of lipids that have been identified as biomarkers or therapeutic targets in adipose tissue lipidomics studies. Finally, the third section introduces mechanistic studies that have investigated the function of enzymes and have elucidated the underlying mechanisms responsible for lipidomic changes.

## Lipidome remodeling of adipose tissue related to various physiological and pathological states

This section summarizes recent investigations on the effects of physiological and pathological stimuli, including aging, sex, exercise, cold exposure, T2D medications, and obesity, on the adipose tissue lipidome. Studies have revealed that adipose tissue lipids are remodeled dynamically in response to different stimuli, establishing links between lipidome changes and various states. A summary of the literature discussed in this section is provided in Table 1 (rodent model studies) and Table 2 (clinical studies).

**Table 1:** Summary of adipose tissue lipidomics studies in rodents.

Category	Subjects studied	Experimental interventions or conditions (sample size)	Sample type		Lipidomic findings	Refs.
Aging	male C57BL/6J mice	25 months (*n* = 3) vs. 2.5 months (*n* = 3) 15 months (*n* = 5) vs. 2 months (*n* = 5)	BAT	↑	PUFA-containing PC, PUFA-containing PE, and SM 36:1 in 25-month-old mice Cer (18:1, 20:0, 22:0, 24:1) in 15-month-old mice	^5^
			iWAT	↑	Cer (18:1, 20:0) in 15-month-old mice	
	*n* = 48, male C57BL/6 mice	24 months (*n* = 24) vs. 2 months (*n* = 24)	BAT	↑	BMP in 24-month-old mice	^7^
			gWAT	↑	BMP, PE, PI, PC O-, PE O-, and LPE O- in 24-month-old mice	
	*n* = 12, male C57BL/6J mice	10–12 months Veh (*n* = 6) vs. 2–3 months Veh (*n* = 6)	BAT mitochondria	↑	Cardiolipin, PC, PE, PI, and PS in 10–12-month-old mice	^15^
			iWAT mitochondria	↑	PC, PE, PI, PS, ePC, ePE, and LPC in 10–12-month-old mice	
			gWAT mitochondria	↑	PC, PS, ePC, and ePE in 10–12-month-old mice	
	*n* = 4–6 per group, male Wistar rats	24 months (*n* = 4–6) vs. 3 months (*n* = 4–6)	gWAT	↑	Cer (16:0, 20:0) in 24-month-old rats	^8^
				↓	Cer 24:1 in 24-month-old rats	
			gWAT plasma membrane	↑	UFA-containing PC, UFA-containing PC O-, ePC, and SM in 24-month-old rats	
Sex	*n* = 12, C57BL/6N mice	Male (*n* = 6) vs. Female (*n* = 6)	BAT	↑	16:0-containing phospholipids, 16:1-containing phospholipids, and 18:2-containing phospholipids in males	^18^
				↓	Phospholipids, 18:0-containing phospholipids, 20:4-containing phospholipids, PG, PI, and SM in males	
	*n* = 11, C57BL/6 mice	Male (*n* = 5) vs. Female (*n* = 6)	BAT	↓	SM in males	^24^
	*n* = 24, C57BL/6 mice	Male sedentary WT/ND (*n* = 12) vs. Female sedentary WT/ND (*n* = 12)	BAT	↓	Cardiolipin 72:8, PC 18:0/20:4, PE 18:0/20:4, and LPC in males	^19^
	*n* = 12, C57BL/6J mice	HF diet (60 kcal% from fat, 6 weeks) Male HF PBS (*n* = 6) vs. Female HF PBS (*n* = 6)	gWAT	↑	PC, PE, PG, PI, PS, LPC, and Cer in HF diet-fed males	^23^
Exercise	*n* = 12, male C57BL/6 mice	Exercise (voluntary wheel running, 3 weeks) Exercise (*n* = 6) vs. Sedentary (*n* = 6)	BAT	↑	PC, PE (40:5, 40:6, 44:4), PC O- (28:2, 36:2), PE O- (34:1, 36:5, 40:6), and LPE 20:1 in exercised mice	^38^
				↓	TG, cardiolipin, PE (24:1, 34:0), PE 44:7/PE O- 44:0, PE-bound MUFAs, PS (16:0/16:1), and LPG in exercised mice	
			iWAT	↑	PE-bound MUFAs in exercised mice	
				↓	TG, acyl chain (44-58 carbons)-containing TG, TG-bound PUFAs, TG-bound short- and medium-acyl chain, PA (16:0/20:4, 18:1/20:2), even-chain fatty acyl (30-36 carbon)-containing PC, PE (34:0, 36:6, 42:4), PE 42:0/PE O- 42:7, PS, PS/LPS-bound 18:2, PS/LPS-bound 20:4, PS/LPS-bound PUFAs, 16:0-containing PS, 18:1-containing PS, 18:2-containing PS, LPG, and LPI in exercised mice	
	*n* = 35, male Wistar rats	Exercise (voluntary wheel running, 8 weeks) Exercise (*n* = 20) vs. Sedentary (*n* = 15)	iWAT	↓	TG in exercised rats	^37^
			gWAT	↑	TG-bound PUFAs, TG-bound omega-6 fatty acids, TG-bound 18:0, and TG-bound 18:2 in exercised rats	
				↓	TG-bound MUFAs, TG-bound 16:0, TG-bound 16:1, and TG-bound 18:1 in exercised rats	
	*n* = 3–4 per group, male Sprague‒Dawley rats	HF diet (71 kcal% from fat, 17 weeks) Exercise (treadmill running, 5 days/week for 8 weeks, 15 min/day at 15 m/min up to 60 min/day at 25 m/min for the last 4 weeks) HET, SET vs. HS vs. SS (*n* = 3–4/group)	gWAT	↑	TG-bound 18:2 in HET compared to HS	^41^
				↓	TG-bound 16:0, 16:1, and 18:1 in SET compared to SS	
Cold exposure	*n* = 16, male C57BL/6J mice	Cold (4 °C, 7 days) Thermoneutral (30 °C, 7 days) Cold (*n* = 8) vs. Thermoneutral (*n* = 8)	BAT	↑	16:0-containing PG, 16:1-containing PG, 18:1-containing PG, 18:2-containing PG, and (PG species with 16:0, 16:1, 18:1 acyl chain)-containing cardiolipin in cold-exposed mice	^61^
			iWAT	↑	16:0-containing PG, 16:1-containing PG, 18:1-containing PG, and 18:2-containing PG (PG species with 16:0, 16:1, 18:1 acyl chain)-containing cardiolipin in cold-exposed mice	
	*n* = 10, male C57BL/6J mice	Cold (4 °C, 3 days) Cold (*n* = 5) vs. RT (*n* = 5)	iWAT	↑	Acylcarnitine, phospholipid-bound 20:4, phospholipid-bound 20:5, phospholipid-bound 22:6, cardiolipin, PC, PE, PI, PS, LPC, LPE, LPG, LPI, Cer, and SM in cold-exposed mice	^59^
	*n* = 10, male C57BL/6J mice	Cold (4 °C, 3 days) Cold (*n* = 5) vs. RT (*n* = 5)	BAT	↑	PC-bound 18:0, PE-bound 18:0, PE-bound 18:2, PS-bound 18:2, LPE-bound 18:0, and LPE-bound 18:1 in cold-exposed mice	^48^
				↓	Phospholipid-bound 16:1, 16:1-containing PE, 16:1-containing PC, and 16:1-containing LPC in cold-exposed mice	
β3-Adrenergic receptor agonist	*n* = 10, male C57BL/6J mice	CL316,243 (1 mg/kg/d, 10 days) CL (*n* = 5) vs. Veh (*n* = 5)	iWAT	↑	Cardiolipin, PC (34:1, 34:2, 36:1, 36:2, 36:3), PE (36:2, 36:3, 38:4, 38:5), and LPC (18:0, 18:1, 18:2) in CL-treated mice	^65^
			gWAT	↑	Cardiolipin, PA, PC (34:1, 34:2, 36:1, 36:2, 36:3, 36:4), PE (34:1, 34:2, 36:2, 36:3, 36:4, 38:4, 38:5), LPC (18:0, 18:1, 18:2), Cer (d18:0/20:0, d18:1/20:0, d18:1/22:0, d18:1/24:0, d18:1/24:1), GluCer (d18:1/18:0, d18:1/20:0, d18:1/22:0, d18:0/24:1), and SM in CL-treated mice	
	*n* = 10, male C57BL/6J mice	CL316,243 (1 mg/kg/d, 3 days) CL (*n* = 5) vs. Control (*n* = 5)	iWAT	↓	Cer, dihydroCer, SM, and sphinganine in CL-treated mice	^69^
			gWAT	↓	Cer, dihydroCer, SM, and sphinganine in CL-treated mice	
	*n* = 12, male C57BL/6J mice	Young (2–3 months old) CL316,243 (1 mg/kg/d, 7 days) Young CL (*n* = 6) vs. Young Veh (*n* = 6)	BAT mitochondria	↑	Cardiolipin, PA, PC (34:1, 34:2, 36:1, 36:2, 36:3, 36:4, 38:4), PE, LPC (16:0, 18:0, 18:2), and very-long acyl chain (22:0, 22:1)-containing sphingolipid in CL-treated mice	^15^
			iWAT mitochondria	↑	Cardiolipin (72:6, 72:8, 74:9, 74:11), PC (34:1, 34:2, 36:1, 36:2, 36:3, 36:4, 38:4), and PE (36:2, 36:3, 38:4) in CL-treated mice	
				↓	PA and SM (16:0, 22:0, 24:1) in CL-treated mice	
			gWAT mitochondria	↑	Cardiolipin, PC (34:1, 34:2, 36:1, 36:2, 36:3, 36:4, 38:4), and PE (34:2, 36:2, 36:3, 36:4, 38:4, 38:5, 38:6, 40:7) in CL-treated mice	
				↓	PA and SM (16:0, 22:0, 24:1) in CL-treated mice	
Anti-diabetic drug	*n* = 16, male C57BL/6J mice	HF diet (60 kcal% from fat, 16 or 32 weeks) Beinaglutide (150 μg/kg/day, 6 weeks) HF-Beinaglutide (*n* = 8) vs. HF-Veh (*n* = 8)	BAT	↓	PI and acyl chain (16-24 carbon)-containing Cer d18:1 in beinaglutide-treated mice	^75^
			iWAT	↑	Acyl chain (>33 carbon)-containing SM in beinaglutide-treated mice	
				↓	PI and acyl chain (16-24 carbon)-containing Cer d18:1 in beinaglutide-treated mice	
			gWAT	↑	Acyl chain (>33 carbon)-containing SM in beinaglutide-treated mice	
				↓	PI in beinaglutide-treated mice	
	*n* = 12, male *db/db* mice	Liraglutide (200 μg/kg/d, 8 weeks) Liraglutide (*n* = 6) vs. Veh (*n* = 6)	BAT	↑	PE 38:6, PC 36:4-1, Cer 40:1-1, Cer 40:2-3, Cer 44:2-3, and SM 42:5 in liraglutide-treated mice	^76^
	*n* = 30, male Zucker Diabetic Fatty rats	Empagliflozin (30 mg/kg/day, 6 weeks) Empagliflozin (*n* = 15) vs. Veh (*n* = 15)	iWAT	↑	PC 40:0 and PC P-16:0/20:4 in empagliflozin-treated mice	^79^
				↓	PC (0:0/16:1, 0:0/18:1, 0:0/20:3), PE (18:2/0:0, 20:4/0:0, 0:0/18:1, 0:0/18:2), LPC (16:1), and LPI (18:1, 20:4) in empagliflozin-treated mice	
			gWAT	↑	DG, 9,10-DiHOME, 12,13-DiHOME, 13-OxoODE, FFA 18:2, and FFA 20:1 in empagliflozin-treated mice	
Time- restricted feeding	*n* = 28, male C57BL/6 J mice	HF diet-AL (48% of energy from fat, ad libitum) HF diet-TRF (48% of energy from fat, restricted to feeding for 12 h per day during the dark phase) HF-TRF (*n* = 14) vs. HF-AL (*n* = 14)	gWAT	↑	SFA (12:0, 16:0, 18:0, 20:0, 22:0) in HF-TRF	^82^
				↓	14-Methyl palmitate in HF-TRF	
Obesity	*n* = 16, male C57BL/6J mice	HF diet (60 kcal% from fat, 8 weeks) HF (*n* = 8) vs. ND (*n* = 8)	BAT	↓	PUFA-containing PE P- (16:0/20:4, 18:1/20:4), LPC P- 18:0, and LPE P- (16:0, 18:1, 18:2, 20:0) in HF	^94^
			iWAT	↓	PUFA-containing PE P- (16:0/20:4, 18:1/20:4), LPC P- 18:0, and LPE P- (16:0, 18:0, 18:1, 18:2, 20:0) in HF	
			gWAT	↓	PUFA-containing PE P- (16:0/20:4, 18:1/20:4) and LPE P- (16:0, 18:0, 18:1, 18:2, 20:0) in HF	
	*n* = 8, male C57BL/6J mice	HF diet (60 kcal% from fat, 12 weeks) HF (*n* = 4) vs. ND (*n* = 4)	BAT	↑	Cer 16:0 in HF	^100^
	*n* = 16, male C57BL/6N mice	HF diet (60 kcal% from fat, 14 weeks) HF (*n* = 8) vs. ND (*n* = 8)	gWAT	↑	Cer (16:0, 18:0) in HF	^101^
	*n* = 13, male WKAH/HkmS1c rats	HF diet (230 g of lard added to the normal diet per kg in place of the dextrin, 8 weeks) HF (*n* = 7) vs. ND (*n* = 6)	gWAT	↑	FFA 18:0 in HF	^91^
				↓	Free PUFA (22:2, 22:4, 22:5) in HF	
	*n* = 10, male C57BL/6J mice	HF diet (60 kcal% from fat, 15 weeks) HF (*n* = 5) vs. ND (*n* = 5)	gWAT	↑	Phospholipid-bound 18:0, sphingolipid-bound 18:0, and dihydroCer in HF	^92^
			gWAT adipocyte derived extracellular vesicles	↓	Cer in HF	
	*n* = 8, male B6.Cg-Lepob/J mice	*ob/ob* (*n* = 4) vs. Lean (*n* = 4)	gWAT	↑	Phospholipid-bound 18:0 and sphingolipid-bound 18:0 in *ob/ob*	
	*n* = 18, male B6.Cg-Lepob/J mice	*ob/ob* (*n* = 8) vs. Lean (*n* = 10)	gWAT adipocyte derived extracellular vesicles	↓	Cer in *ob/ob*	

Elevated levels of lipid species corresponding to distinct physiological conditions are denoted by an upward arrow (↑), whereas reduced levels of lipid species are represented by a downward arrow (↓).

*AL* Ad libitum, *BAT* brown adipose tissue, *BMP* bis(monoacylglycero)phosphate, *Cer* ceramide, *CL* CL316,243, *DG* diacylglycerol, *ePC* ether-linked PC, *ePE* ether-linked PE, *FFA* free fatty acid, *GluCer* glucosylceramide, *gWAT* gonadal white adipose tissue, *HET* high-fat diet endurance training, *HF* high fat, *HS* high-fat diet sedentary, *iWAT* inguinal white adipose tissue, *LPC* lysophosphatidylcholine, *LPC P-* plasmenyl LPC, *LPE* lysophosphatidylethanolamine, *LPE O-* plasmanyl LPE, *LPE P-* plasmenyl LPE, *LPG* lysophosphatidylglycerol, *LPI* lysophosphatidylinositol, *LPS* lysophosphatidylserine, *MUFA* monounsaturated fatty acid, *ND* normal diet, *PA* phosphatidic acid, *PBS* phosphate-buffered saline, *PC* phosphatidylcholine, *PC O-* plasmanyl PC, *PC P-* plasmenyl PC, *PE* phosphatidylethanolamine, *PE O-* plasmanyl PE, *PE P-* plasmenyl PE, *PG* phosphatidylglycerol, *PI* phosphatidylinositol, *PS* phosphatidylserine, *PUFA* polyunsaturated fatty acid, *RT* room temperature, *SET* standard-diet endurance training, *SFA* saturated fatty acid, *SM* sphingomyelin, *SS* standard-diet sedentary, *TG* triglyceride/triacylglycerol, *TRF* time restricted feeding, *UFA* unsaturated fatty acid, *Veh* vehicle, *WT* wild type.

**Table 2:** Summary of adipose tissue lipidomics studies in humans.

Category	Subjects studied	Experimental interventions or conditions (sample size)	Sample type		Lipidomic findings	Refs.
Exercise	*n* = 36, male subjects	Exercise (4 months of endurance training and then gradually changed to interval training, 7 months) Exercise (*n* = 15) vs. Sedentary (*n* = 21)	SAT	↑	Fatty acid 18:0 in exercised subjects	^42^
				↓	Fatty acid 16:1 in exercised subjects	
	*n* = 46, NAFLD patients	Exercise (high-intensity interval training based on the ergospirometry test, 12 weeks) Exercise (*n* = 21) vs. Sedentary (*n* = 25)	SAT	↑	LPE (16:0, 18:0) in exercised NAFLD patients	^49^
	*n* = 72, male/female overweight elderly subjects	Exercise (daily physical activity level of at least 30 min of moderate intensity and included both aerobic and strength training, 6 months) Exercise (*n* = 43) vs. Sedentary (*n* = 30)	SAT	↑	Fatty acid 18:2, omega-6 PUFAs in exercised overweight elderly subjects	^39^
	*n* = 27, female subjects aged 65–80	Exercise (combined aerobic and resistance training, 1 h, 3 times a week, 4 months) After Exercise+Placebo vs. Before Exercise+Placebo	SAT	↑	Total PAHSA, 5-PAHSA, 9-PAHSA, 10-PAHSA, 11-PAHSA in exercised elderly women	^53^
Anti-diabetic drugs	*n* = 7, male/female T2D patients	Pioglitazone (45 mg/day, 6 months) Pioglitazone (*n* = 7) vs. Veh (*n* = 0)	SAT	↑	SFA-containing phospholipids in pioglitazone-treated patients	^72^
				↓	Free AA, cardiolipin, AA-containing PE P-, and AA-containing phospholipids in pioglitazone-treated patients	
Obesity	*n* = 86, male/female subjects	Lean (BMI 21.6–24.6 kg/m^2^; age = 68 ± 10.9 years; male/female = 3/2) Obesity (BMI 43.9–46.3 kg/m^2^; age = 45 ± 2.2 years; male/female = 26/55) Obesity (*n* = 81) vs. Lean (*n* = 5)	SAT	↑	PUFA (20:4, 20:5, 22:5, 22:6)-containing TG, PUFA (20:4, 20:5, 22:6)-containing PC P-, and sphingadiene Cer (SPB 18:2;O2) in obesity	^2^
				↓	MUFA-containing TG and SFA-containing TG in obesity	
			VAT	↑	PUFA (20:4, 20:5, 22:5, 22:6)-containing TG, C18 acyl chain-containing PE P-, and sphingadiene Cer (SPB 18:2;O2) in obesity	
				↓	MUFA-containing TG and SFA-containing TG in obesity	
	*n* = 53, male/female subjects	Lean (BMI 24.4–26.22 kg/m^2^) Obesity (BMI 41.59–44.73 kg/m^2^) Pathogenic obesity (BMI 46.87–50.31 kg/m^2^) Pathogenic obesity (*n* = 18) vs. Obesity (*n* = 18) vs. Lean (*n* = 17)	VAT	↑	PE P- (16:0/20:4) and LPE P- (16:0, 18:0, 18:1) in pathogenic obesity compared to obesity or lean PC P- (16:0/16:0, 16:0/20:4) and PE P- (18:0/20:4) in pathogenic obesity compared to obesity	^95^
				↓	LPE P-18:0 in obesity compared to lean	
	*n* = 13 pairs of monozygotic twins *n* = 8 pathogenic obese subjects	Low BMI (BMI < 25 kg/m^2^) High BMI (BMI ≥ 25 kg/m^2^) Pathogenic obesity (BMI 47.0–60.4 kg/m^2^) BMI discordant twin pairs (*n* = 13; 15.2 kg (20%, 5.3 kg/m^2^) heavier than the nonobese twin) Pathogenic obesity (*n* = 8)	SAT	↑	AA-containing PE P- in obesity compared to lean	^86^
				↓	PE P- (16:0/20:4, 18:1/20:4) in pathogenic obesity compared to obesity or lean	
			VAT	↓	PE P- (16:0/20:4, 18:1/20:4) in pathogenic obesity compared to obesity or lean	
	*n* = 20 subjects	Lean (BMI < 25 kg/m^2^) Obesity (BMI > 30 kg/m^2^) Obesity (*n* = 10) vs. Lean (*n* = 10)	VAT	↑	Cer (14:0, 16:0, 16:1, 18:0, 18:1, 22:1) in obesity	^101^
	*n* = 71, male/female subjects	Nonobesity (BMI 18.5–26.9 kg/m^2^) Morbid obesity (BMI > 40 kg/m^2^) Low IR or IS state (FG < 100 mg/dL and HOMA-IR < 2.5) High IR state (FG levels 100–125 mg/dL or HOMA-IR > 3.4) Obesity with low IR (*n* = 11) vs. Obesity with high IR (*n* = 25)	VAT	↑	C18 acyl chain-containing phospholipid and, LPE 18:2 in IS obesity compared to IR obesity	^93^
	*n* = 48, male/female subjects	BMI 17.96–27.03 kg/m^2^	SAT	↑	PUFA-containing PC P- and PUFA-containing PE P- *positive correlation with BMI	^94^
				↓	LPE P- 18:0 *negative correlation with BMI	

Elevated levels of lipid species corresponding to distinct physiological conditions are denoted by an upward arrow (↑), whereas reduced levels of lipid species are represented by a downward arrow (↓).

*AA* arachidonic acid, *BMI* body mass index, *Cer* ceramide, *FG* fasting plasma glucose, *IR* insulin resistance, *IS* insulin sensitive, *LPE* lysophosphatidylethanolamine, *LPE P-* plasmenyl LPE, *MUFA* monounsaturated fatty acid, *NAFLD* nonalcoholic fatty liver disease, *PAHSA* palmitic acid esters of hydroxystearic acid, *PC P-* plasmenyl PC, *PE P-* plasmenyl PE, *PUFA* polyunsaturated fatty acid, *SAT* subcutaneous adipose tissue, *SFA* saturated fatty acid, *TG* triglyceride/triacylglycerol, *T2D* type 2 diabetes, *VAT* visceral adipose tissue, *Veh* vehicle.

### Aging

Aging is accompanied by changes in adipocyte size and adipose tissue mass, as well as lipid composition remodeling in adipose tissue^3,4^. For instance, there is an increase in adipocyte size from middle to old age, followed by a decrease in size in advanced age (~30 months) in mice^3^. The composition of lipids also indicates the extent of age-related changes. In this section, we have summarized lipidomics studies that investigated the remodeling of adipose tissue in mice with respect to aging.

The composition of phospholipids undergoes significant changes with aging. Gohlke et al.^5^ analyzed the lipidome profile in brown adipose tissue (BAT) and inguinal white adipose tissue (iWAT) of mice ranging from 2 to 25 months of age. The authors found a positive correlation between aging and the content of phospholipids in BAT, particularly phosphatidylethanolamine (PE) and phosphatidylcholine (PC) containing polyunsaturated fatty acids (PUFAs). The levels of these phospholipids gradually increase in BAT as aging progresses, peaking at 25 months of age^5^. This is consistent with the age-induced elevation of highly unsaturated fatty acids in membrane lipids, which can render them more susceptible to oxidative damage^6^. Other studies in rodents have revealed that aging elevates PE, phosphatidylinositol (PI), and ether-phospholipids such as plasmanyl phosphatidylcholine (PC O-), plasmanyl phosphatidylethanolamine (PE O-), and plasmanyl lysophosphatidylethanolamine (LPE O-) in the gonadal white adipose tissue (gWAT) of 24-month-old male mice^7^ and total ether-linked PC (ePC) levels as well as unsaturated fatty acid-containing PC and PC O- in the gWAT plasma membrane of 24-month-old male rats^8^. In line with the increase in various phospholipid species in murine adipose tissue, the GOLDN study in which 980 human subjects aged 18–87 years participated revealed that the content of specific phospholipids, including PC, PE, PI, phosphatidylglycerol (PG), and LPE, significantly increases in plasma with age^9^. Another human study with northern Italian centenarians demonstrated an increase in PUFA-containing PC levels in the serum of elderly individuals aged 56–86^10^. Studies have demonstrated that aging enhances the levels of bis(monoacylglycero)phosphate, a specific subclass of phospholipids found in acidic organelles such as lysosomes and late endosomes, in the BAT and gWAT of 24-month-old male mice compared to 2-month-old male mice^7^.

Aging also induces significant changes in sphingolipid profiles. The defect in the thermogenic capacity of adipose tissue due to aging has been attributed to the accumulation of ceramide (Cer) in adipose tissue, which inhibits differentiation into brown adipocytes and their mitochondrial respiration^5^. In line with this, targeted lipidomics analysis of ceramide revealed upregulation of Cer (18:1, 20:0, 22:0, and 24:1) levels in BAT and Cer (18:1 and 20:0) levels in iWAT of 15-month-old male mice compared to 2-month-old male mice. In a study investigating the aging-induced alterations in the lipidome of gWAT, it was found that the levels of Cer (16:0 and 20:0) are elevated, while Cer 24:1 is decreased in gWAT of 24-month-old male rats compared to 3-month-old male rats^8^. The increased expression of enzymes involved in ceramide biosynthesis, such as serine palmitoyltransferase (SPT) and ceramide synthase 6 (CerS6, also known as LASS6), is likely responsible for the elevated ceramide species, including Cer 16:0, observed during aging^8^. In addition to Cer, sphingomyelin (SM), another type of sphingolipid, also undergoes changes in abundance with aging. For example, SM 36:1 is elevated with aging in mouse BAT and shows the highest level at 25 months of age^5^. Total SM is increased in the gWAT plasma membrane of rats at 24 months of age^8^. The increased levels of SM observed in BAT and gWAT during aging may serve to enhance mechanical resistance, as SM is known to reduce membrane fluidity^11^. Human plasma lipidomics studies with elderly subjects have also reported elevated levels of total Cer^9^, along with an increase in SM (16:0 and 24:1)^10^ or total SM levels^9^. Higher levels of sphingolipids in human plasma have been proposed as biomarkers for Alzheimer’s disease and insulin resistance^12,13^. Plasma Cer 16:0 has also been identified as a potential biomarker of aging^14^. Considering that the changes in the adipose tissue lipidome during aging in mouse models are reflected in human plasma levels, we speculate that adipose tissue lipid remodeling during aging is a significant contributor to the observed alterations in plasma lipid composition. Further investigation is necessary to establish the clinical relevance of the findings from adipose tissue lipidomics studies using aged rodent models.

Various phospholipid classes are also found within the mitochondrial membrane, aiding in the maintenance of structural integrity and the activity of mitochondrial membrane proteins. Consequently, Rajakumari et al.^15^ performed mitochondrial lipidome analysis in BAT and WAT of young (2–3 months old) and middle-aged (10–12 months old) mice to investigate age-induced mitochondrial lipid remodeling. Aging increased the total contents of lysophosphatidylcholine (LPC), PC, PE, PI, phosphatidylserine (PS), ePC, and ether-linked PE (ePE) in iWAT; the total contents and major species of PC, PS, ePC, and ePE in gWAT; and the total PC, PE, PI, PS, and cardiolipin levels in the mitochondrial fraction of BAT^15^.

### Sex

Sex differences have direct or indirect effects on a wide range of physiological and pathological functions. There are well-established biological differences between the sexes in adipose tissue, such as differences in anatomical distribution and hormone responsiveness^16^. Similarly, sex-based differences in lipid and lipoprotein metabolism have been identified, including variations in cholesterol synthesis, clearance, and transport, which can contribute to distinct lipid profiles^17^.

Hoene et al. analyzed the sex dimorphism of the phospholipid composition in BAT and WAT of male and female mice^18^. In this study, total phospholipid levels were higher in female BAT than in male BAT, with a significant increase in PG and PI levels and an increasing trend in PE^18^. This is further supported by the study conducted by Tóth et al., who reported a significantly higher ratio of membrane lipids to triglycerides (TGs) in female BAT^19^. In line with studies indicating that estrogen promotes the conversion of linoleic acid to arachidonic acid (AA)^20,21^, female BAT exhibits higher levels of stearic acid (18:0)- and AA (20:4, n-6)-containing phospholipids compared to those in males^18,19^. Conversely, female BAT displays lower levels of palmitic acid (16:0)-, palmitoleic acid (16:1, n-7)-, and linoleic acid (18:2, n-6)-containing phospholipids compared to those in males^18^, possibly reflecting the influence of sex hormones. The clinical data indicated a higher concentration of AA-containing phospholipids such as PE O- (16:1/20:4 and 18:2/20:4) in the plasma of women than men^22^, suggesting the clinical relevance of the findings in mouse studies. In addition, Tóth et al.^19^ observed that major cardiolipin species, cardiolipin 72:8, and LPC levels are also significantly greater in the BAT of female mice. In the obese state, however, the contents of PS, PC, LPC, PE, PG, and PI are higher in the gWAT of male mice than in that of females^23^. In terms of sphingolipids, several studies in mice have reported that SM is higher in female BAT than in male BAT^18,24^. Another study examining sphingolipid levels in the gWAT of obese mouse models reported increased ceramide levels in the gWAT of obese male mice compared to that of obese female mice^23^.

It is noteworthy that ceramide is strongly associated with metabolic disorders^25^, while SM, PC, and LPC are also correlated with obesity and insulin resistance^26^. Therefore, these differences in lipid composition between the sexes are consistent with the sex-specific differences in susceptibility to metabolic disorders^27^. Additionally, the sex dimorphism of phospholipids may also be attributed to the differential expression and activity of phospholipid metabolic enzymes between the sexes^28^, along with the sex-dependent variations in mitochondrial size and cristae density^29^. This can serve as evidence for the higher thermogenic capacity in adipose tissue and subsequent protection against obesity-related metabolic diseases in female mice^30^.

### Exercise

Regular exercise has been shown to increase lifespan by improving overall muscle strength and endurance and by protecting against a range of diseases^31,32^. The beneficial effects of exercise are attributed to its ability to induce an adaptive response, including alterations in lipid metabolism^33^ and remodeling of adipose tissue^34^. Exercise can be categorized into various types based on factors such as repetition, intensity, and other criteria. In experimental models, acute exercise refers to a single performance, whereas chronic exercise refers to repeated performance^35^. Endurance training involves the repetitive contraction of muscles against submaximal resistance over an extended duration, whereas strength training involves the application of maximal force within a shorter timeframe^36^. In this review, our focus is on studies that employed chronic exercise and endurance training as interventions in their experimental models.

A decrease in total TGs is observed in both murine iWAT^37,38^ and BAT^38^ in response to increased energy demand during exercise. May et al.^38^ reported a significant reduction in TG species with chain lengths of 44–58 carbons, as well as in PUFAs and short- and medium-acyl chains incorporated into TGs in iWAT after three weeks of voluntary wheel running. Conversely, a previous study by Petridou et al., which had a longer training period, demonstrated increased PUFA and omega-6 contents, as well as a decreased monounsaturated fatty acid (MUFA) content in TGs in the gWAT of rats after 8 weeks of voluntary wheel running^37^. A clinical study reported that a 6-month moderate-intensity physical activity regimen, comprising both aerobic and strength training, elevated the omega-6 PUFA content^39^. This elevation was primarily driven by an increase in linoleic acid in the subcutaneous adipose tissue (SAT) of overweight elderly subjects^39^. The majority of previous studies have demonstrated higher levels of PUFAs and omega-6 fatty acids in the adipose tissue of trained animals and humans^40^. Additionally, after 8 weeks of endurance training on a treadmill, there was a decrease in TG-bound palmitic acid, palmitoleic acid, and vaccenic acid (18:1, n-7) in the gWAT of the standard diet-fed group and an increase in TG-bound linoleic acid in the gWAT of the high-fat (HF) diet-fed group^41^. A previous study also demonstrated a decrease in the levels of TG-bound fatty acyl chain 16:0, 16:1, and 18:1 and an increase in TG-bound fatty acyl chain 18:0 and 18:2 in the gWAT of exercised rats^37^. In another clinical study investigating the fatty acid profiles of trained human SAT, it was found that the content of palmitoleic acid decreases, while that of stearic acid increases after 7 months of endurance and interval training^42^.

Chronic exercise leads to substantial changes in phospholipid composition. May et al.^38^ demonstrated that the phospholipid profiles of iWAT and BAT in mice undergo alterations after three weeks of voluntary wheel running. Specifically, there was a decrease in the total levels of PS, lysophosphatidylglycerol (LPG), and LPI in iWAT. In BAT, there is an overall decrease in the abundance of cardiolipins and LPG, while the total levels of PC are elevated. The authors reported that PA (16:0/20:4 and 18:1/20:2) and PS species containing 16:0, 18:1, or 18:2 chains, with the largest decrease in PS 16:0/18:0, are reduced after chronic exercise along with decreased PS/lysophosphatidylserine (LPS)-bound PUFA, linoleic acid, and AA contents in iWAT^38^. A similar pattern of change in the phospholipid fatty acid content is also observed in the skeletal muscle of exercised rats, as long-chain PUFAs and AAs are decreased while linoleic acid is increased^43^. Of the PS species, only PS 16:0/16:1 is significantly decreased in BAT^38^.

Among the PC species, PC species with even chain lengths (30–36 carbons), especially PC 36:4, are reduced after chronic exercise in iWAT, while exercise leads to an increase in the content of PC species with both shorter (28:1 and 30:0) and longer (36:2, 38:6, 40:6, and 44:0) carbon chain lengths as well as PC O- (28:2 and 36:2) in BAT^38^. In line with this, PC 40:6 is also reported to increase in the skeletal muscle of trained rats^44^. This increase in PC species containing docosahexaenoic acid (DHA, 22:6, n-3) may support the positive effects of exercise, as dietary DHA is incorporated into phospholipids and exerts beneficial effects such as reducing the risk of cardiovascular disease^45^ and improving mitochondrial ADP sensitivity^46^. Furthermore, elevated PC 38:6 is associated with a decreased risk of diabetes^47^. PC species with longer carbon chains that are elevated by exercise in BAT differ from those that are increased by cold exposure, such as PC subclasses C18:0 and C18:2^48^, indicating that distinct mechanisms are responsible for the remodeling of PC depending on the physiological state.

Among the PE species, 3 weeks of voluntary wheel running reduces PE (34:0, 36:6, and 42:4) and PE 42:0/PE O- 42:7, whereas it increases the PE-bound MUFA content in iWAT^38^. In contrast, exercise training significantly enhances the levels of PE (40:5, 40:6, and 44:4), LPE 20:1, and PE O- (34:1, 36:5, and 40:6), while PE (24:1 and 34:0), PE 44:7/PE O- 44:0, and PE-bound MUFA levels are decreased in BAT^38^. In patients with nonalcoholic fatty liver disease (NAFLD), 12 weeks of high-intensity interval training leads to an increase in the levels of LPE (16:0 and 18:0) in SAT and plasma^49^.

Interestingly, these PE and PC species that are elevated in BAT do not overlap with the species that are decreased in iWAT after chronic exercise. Different aspects of the PC and PE composition may reflect functional discrepancies between BAT and WAT^50^. Indeed, exercise has opposing effects on mitochondrial activity in BAT and iWAT, as it reduces mitochondrial activity in BAT while enhancing it in iWAT^51^. Concomitantly, the levels of BAT markers such as *Ucp1, Cox8b*, and *Cidea* are significantly increased in the iWAT of healthy male mice in response to exercise training, while they are reduced in the BAT of the same group^19^, suggesting browning of iWAT, which is a well-established exercise-induced effect on adipose tissue^52^.

Several studies have investigated the changes in palmitic acid esters of the hydroxystearic acid (PAHSA) family in response to exercise in humans. Brezinova et al.^53^ demonstrated that 4 months of combined aerobic and resistance training elevates the content of the PAHSA family in the adipose tissue of elderly women, including 5-PAHSA, 9-PAHSA, 10-PAHSA, 11-PAHSA, and their total levels. PAHSA is a member of the fatty acyl esters of hydroxy fatty acids (FAHFA) family that exerts antidiabetic effects^54^. Thus, exercise-induced PAHSAs might account for the lipid-mediated beneficial effect of exercise on whole-body insulin sensitivity^53^. Furthermore, exercise leads to an increase in TG estolides, which were shown to be a major reservoir for FAHFA in the same study^53^.

### Cold exposure/β3-adrenergic receptor agonists

In mammals, BAT plays a crucial role in protecting against hypothermia through the activation of nonshivering thermogenesis^55^. Similarly, WAT, which serves as the primary energy storage organ in mammals, can undergo thermogenic beige adipocyte recruitment, a phenomenon referred to as browning. Activation of BAT and browning of WAT can be triggered by various stimuli, such as exposure to cold temperature or β3-adrenergic receptor agonists^56,57^. Given their thermogenic energy-dissipating properties, brown/beige adipocytes represent a promising therapeutic target for the treatment of obesity^58^.

A recent lipidomics study demonstrated that 3 days of cold exposure increases the levels of acylcarnitine in iWAT^59^. Simcox et al.^60^ reported that cold exposure elevates the level of acylcarnitine, which is used as a fuel source in BAT during thermogenesis, in the circulation and upregulates hepatic *Cpt1a/b* genes that are involved in acylcarnitine metabolism. Given that browning of iWAT is observed after 3 days of cold exposure^59^, it is plausible that acylcarnitine is utilized for thermogenesis in cold-exposed iWAT, similar to BAT.

Among phospholipids, cardiolipin, LPC, LPE, LPG, LPI, PC, PE, PI, and PS are increased in iWAT after 3 days of cold exposure^59^. Lynes et al.^61^ confirmed that cold exposure activates the cardiolipin biosynthetic pathway in both brown and beige fat. Cardiolipin, a major component of the inner mitochondrial membrane, plays a crucial role in mitochondrial biogenesis and function^62–64^. PG is a precursor phospholipid used for synthesizing cardiolipin^64^. In line with the correlation of these two lipids, species-specific elevations of PGs with fatty acyl chains 16:0, 16:1, 18:1, and 18:2 and cardiolipins containing these PGs are observed in BAT and iWAT after cold exposure^59,61^. Human subjects exposed to mild cold for 1 h also exhibit an increase in several PG and LPG species, such as PG 20:0/22:5 and LPG (18:0 and 18:1), in serum^61^. Analysis of the total pool of phospholipid-bound fatty acyl chains revealed that PUFAs, including DHA, eicosapentaenoic acid (EPA, 20:5, n-3), and AA, are significantly increased in cold-challenged iWAT^59^. According to the study of Hoene et al.^18^, more than half of the top eleven BAT-specific lipids are DHA-enriched phospholipids. In BAT, cold exposure alleviates the levels of 16:1-containing PE, PC, and LPC, thus reducing the total 16:1 acyl chain level in phospholipids, which may be due to decreased expression of stearoyl-CoA desaturase 1 (*Scd1*), the gene responsible for desaturation of palmitic and stearic acid^48^. In addition, the elevation of phospholipid acyl chains such as PC-bound 18:0, PE-bound 18:0 and 18:2, PS-bound 18:2, and LPE-bound 18:0 and 18:1 also indicates that cold exposure remodels the phospholipid composition in BAT^48^.

After a 3-day cold exposure, there were elevated levels of Cer and SM in iWAT^59^. This increase is accompanied by the upregulation of genes involved in sphingomyelin biogenesis (*Sptlc1, Cers4*, and *Degs2*) and downregulation of sphingolipid breakdown-related genes (*Asah1, Asah2, Acer3, Sphk1*, and *Sgpp1*)^59^.

The browning of WAT can be induced by β-adrenergic stimuli, but visceral adipose tissue (VAT) is less sensitive than SAT. To investigate this phenomenon in relation to lipid remodeling, changes in the lipidome of iWAT and gWAT in mice were examined following pharmacological stimulation of the β3-adrenergic receptor^65^. Treatment with the β3-adrenergic receptor agonist CL316,243 (1 mg/kg/day, 10 days) increases the contents of PC, PE, LPC, and cardiolipin in both gWAT and iWAT, with a more pronounced effect in gWAT^65^. Phospholipids are major components of cellular and organelle membranes, and PC and PE constitute approximately 70% of mitochondrial phospholipids^66^. Increases in PC and PE have been shown to have physiological functions that alleviate metabolic disorders associated with obesity^67,68^, and their increase may contribute to the phospholipid supply for mitochondrial biogenesis and thermogenesis. CL316,243 treatment leads to a 10-fold increase in cardiolipin levels in gWAT and a 2.5-fold increase in iWAT, with 72:8 (18:2) and 72:7 (18:2) showing the most prominent changes^65^. This increase in cardiolipin levels is supported by a concomitant upregulation in the mRNA expression of cardiolipin synthase 1^65^. For sphingolipids, unexpectedly, CL316,243 treatment results in a significant increase in glucosylceramide and SM in gWAT, including Cer d18:1 species^65^. In contrast to the aforementioned findings from long-term treatment (10 days), the results of a three-day administration of CL316,243 yielded contradictory outcomes^69^. According to Chaurasia et al.^69^ β-adrenergic activators reduce overall adipose ceramide, dihydroceramide, sphinganine, and sphingomyelin levels in iWAT and gWAT without altering BAT, implying important roles of sphingolipid depletion in the thermogenic properties of WAT. These contradictory results may be due to the highly plastic nature of adipose tissue in response to varying pharmacological exposure conditions. Nevertheless, based on the findings from both studies, it is plausible that the brown heterogeneity observed in gWAT and iWAT may be linked to alterations in sphingolipid metabolism.

Rajakumari et al.^15^ investigated the effect of CL316,243 (1 mg/kg/day, 7 days) stimulation on mitochondrial lipid remodeling in the adipose tissue of mice. The phospholipid composition of the mitochondrial membrane is known to play an important role in mitochondrial function^67^. β3-Adrenergic stimuli increase the LPC (16:0, 18:0, and 18:2) content in mouse BAT mitochondria and PC species encompassing 34:1, 34:2, 36:1, 36:2, 36:3, 36:4, and 38:4 in the mitochondria of all adipose depots^15^. CL316,243 elevates the total PE levels by approximately threefold in BAT mitochondria and affects the unsaturation levels, especially increasing the PE 38:4 levels, not only in BAT but in all adipose tissue mitochondria^15^. The increase in mitochondrial PE levels correlates positively with electron transport chain complex activities, ATP levels, and mitochondrial respiration^67,70^. PE accounts for approximately 40% of the phospholipids that make up the mitochondrial membrane, and it is highly likely to be synthesized in a mitochondria-specific manner^70^. Interestingly, recent studies have revealed that the reduction in mitochondrial PE by phosphatidylserine decarboxylase KO lowers UCP1 activity and thermogenesis in BAT. These researchers suggest that loss of PE may alter the lipid bilayer properties of the inner mitochondrial membrane to reduce the protonophoric activity of UCP1, which is ultimately required for thermogenesis in BAT^70^. In terms of sphingolipids, CL316,243 increases the synthesis of very-long-chain fatty acid (22:0 and 22:1)-containing sphingolipids in BAT, while major SM species (16:0, 22:0, and 24:1) are reduced by 2.5- to 5-fold in the mitochondria of iWAT and gWAT^15^. SM accumulation potentially reduces the mitochondrial thermogenic capacity of adipose tissue, and SM enrichment may disrupt proton leakage across the mitochondrial membrane in WAT mitochondria^15,69^.

Overall, the lipidomics analysis of white and brown adipose tissue, both in whole tissue and in the mitochondrial fraction, revealed that lipidome changes induced by CL316,243 treatment followed a similar trend in terms of phospholipids. However, some differences were noted specifically in gWAT. In both whole adipose tissue and the mitochondrial fractions of WAT and BAT, there was an increase in the levels of PE and cardiolipin, which are major components of mitochondrial phospholipids. In contrast, gWAT exhibited a distinct profile for PA, with an overall increase observed at the tissue level but a decrease in the levels within the mitochondria. For sphingolipids in gWAT, contrary results were observed depending on the duration of exposure to CL316,243.

### Anti-diabetic drugs

Various pharmacological agents, including drugs and dietary supplements, have been shown to modulate lipid metabolism and remodeling of adipose tissue^1^. We have summarized recent lipidomics studies investigating the effects of representative T2D therapeutics on the lipid remodeling of adipose tissue.

Pioglitazone, a PPARγ agonist, is a T2D medication^71^, and its action can be partly explained by the redistribution of body fat from VAT and ectopic fat to SAT^72^. Palavicini et al.^72^ analyzed the effect of pioglitazone (45 mg/day) on adipose tissue redistribution in seven obese patients with T2D using class-targeted shotgun lipidomics to examine changes in the SAT lipidome composition. Pioglitazone treatment increased saturated fatty acid (SFA)-containing phospholipids, particularly PE, and decreased AA-containing phospholipids as well as plasmenyl PE (PE P-), free AA, and cardiolipin^72^. The possible mechanism of phospholipid remodeling mediated by pioglitazone is abrogated synthesis of AA accompanied by downregulation of free linoleic acid, an AA precursor lipid^72^.

Another class of T2D therapeutics is GLP-1 receptor agonists^73^, and their effects on the activation of BAT metabolism have been reported^74^. When beinaglutide (150 μg/kg/day, 6 weeks), a human GLP-1 analog, is administered to HF diet-induced obese mice, among the phospholipid subclasses, the greatest changes in PC and PE are observed in adipose tissue^75^. BAT contains higher levels of PE than iWAT and gWAT. In BAT, most of the altered PE species are increased, whereas in WAT, they are decreased^75^. Similarly, altered PC species levels are elevated in BAT but decreased in WAT^75^, revealing different responses of BAT and WAT to the GLP-1 analog. A decrease in the overall levels of PI is observed in all three adipose depots, accompanied by reductions in specific species containing C36 and C38 acyl chains^75^. The total levels of ceramide are decreased in BAT and iWAT, along with an increase in the expression level of *Acer2* in iWAT, one of the ceramidases^75^. Furthermore, the administration of beinaglutide increases the level of SM with acyl chain lengths of more than 33 carbons in WAT^75^, and this effect is also observed during cold exposure^59^. When male leptin receptor-deficient *db/db* mice are treated with liraglutide, another GLP-1 agonist, the levels of 6 out of 128 lipid species, namely, PE 38:6, PC 36:4-1, Cer 40:1-1, Cer 40:2-3, Cer 44:2-3, and SM 42:5, are augmented in BAT^76^. In contrast, when newly diagnosed T2D patients are treated with exenatide for 12 weeks, PC, PE, and Cer levels are decreased in serum, with a reduction in PE 38:6 levels being particularly characteristic^77^.

Sodium-glucose cotransporter 2 (SGLT2) inhibitors are a T2D therapeutic that lowers blood glucose levels by inhibiting renal glucose reabsorption^78^. Recently, a research group investigated the effects of empagliflozin treatment (30 mg/kg/day, 6 weeks) on the lipidome profiles of the WAT of Zucker diabetic fatty rats, a T2D model^79^. This study found that empagliflozin leads to an increase in the levels of oxidized fatty acids, DG, gadoleic acid (20:1, n-6), and linoleic acid (18:2, n-6) in gWAT^79^. They also found that empagliflozin increases the gene expression levels of the major lipases (*Lipe* and *Pnpla2*) in gWAT, providing a potential mechanistic explanation of the lipidome-modifying role of empagliflozin in adipose tissue^79^. On the other hand, the majority of species that underwent significant changes in iWAT are phospholipids. These involve decreased levels of four species of LPE, four species of LPC, and three species of LPI alongside increased levels of PC 40:0 and PC P-16:0/20:4^79^. While it is clear that the effects of empagliflozin include changes in lipidome composition, further investigation is required to understand the molecular players that remodel adipose tissue lipidomes in response to empagliflozin.

### Time-restricted feeding

Among various dietary interventions, time-restricted feeding (TRF) has gained attention in recent years due to its potential to facilitate weight loss and improve metabolic health^80,81^. Mehus et al.^82^ investigated the effects of TRF on adipose tissue lipidome profiles in male mice. When male mice are fed low-fat (LF) or HF diets and allowed to eat freely (ad libitum, AL) or restricted to eating from 7 pm to 7 am (TRF), HF-TRF prevents insulin resistance and hepatic steatosis compared to the HF-AL diet^82^. The respiratory exchange ratio value during the dark phase was higher in the HF-TRF group than in the HF-AL group but lower in the HF-TRF group during the light phase, indicating an increase in the oxidation rate of fatty acids used for energy^82^. The lipidomics results of the gWAT show that the HF-TRF group has increased levels of several SFA species (12:0, 16:0, 18:0, 20:0, and 22:0) compared to levels in the HF-AL group, which could be a result of increased de novo lipogenesis^82^. TRF also increases FFA release from adipocytes^83^, which is consistent with the increase in free SFA levels in plasma^82^. The branched-chain fatty acid 14-methyl palmitate is decreased in the HF-TRF group compared to the HF-AL group^82^. Although the regulatory roles of 14-methyl palmitate were not investigated in this TRF mouse model, mono-methyl branched chain fatty acid (mmBCFA), which is primarily synthesized in adipose tissue, has been reported to have a positive correlation with insulin sensitivity^84^. Consistently, a clinical study demonstrated that abdominal adipose tissue mmBCFA is decreased in obese individuals^85^. While TRF does not have a significant effect on weight loss, as shown in human clinical studies^80,81^, it forms a unique lipid profile and metabolic improvements in the adipose tissue of diet-induced obese mice.

### Obesity

Obesity is a complex metabolic disorder characterized by the excessive accumulation of adipose tissue, and alterations in the adipose tissue lipidome have been linked to the development and progression of obesity and related metabolic disorders^86^.

Lipidome analysis of SAT and VAT in lean or obese individuals has been performed in several studies. VAT lipids are composed of ~90% TG, while phospholipids and sphingolipids account for only 3% of total lipids^87^. However, changes in the phospholipid composition seem to play an important role in the development and prognosis of metabolic diseases^88^. In obese WAT, the abundance of TGs with at least one PUFA residue is increased^2,89^, which correlates with larger lipid droplet size^90^, whereas the abundances of SFA or MUFA residue-containing TG species are decreased in obesity^2,89^. Free PUFAs (22:2, 22:4, and 22:5) are significantly decreased in the gWAT of HF diet-induced obese rats^91^. Incorporating highly unsaturated PUFAs such as FA 20:4 into TGs for storage in adipose tissue can decrease the plasma n-6/n-3 ratio and have a protective role in whole-body metabolism^89^. In rodent studies, most SFA and MUFA levels in FFA analyses of gWAT in HF diet-induced obese rats show no change; however, the content of FFA 18:0 exhibits a positive correlation with obesity^91^. Not only was the unesterified form of C18:0 increased, but there was also an increase observed in the contents of phospholipid and sphingolipid-bound C18:0 acyl chains in both *ob/ob* and HF diet-induced obese mice, particularly in lysophospholipids and sphingolipids^92^.

Interestingly, a clinical study has delved further into the differences in phospholipid profiles between insulin-sensitive and insulin-resistant obese patients. This study identified 18 carbon acyl chain-containing phospholipids in VAT as a major lipid species that differentiates insulin-sensitive individuals and insulin-resistant obese patients^93^. Specifically, it has been observed that higher levels of LPE 18:2 are present in obese groups with greater insulin sensitivity, thereby serving as a distinguishing factor for insulin responsiveness among obese patients^93^.

Lange et al.^2^ deeply investigated the WAT lipidome of 86 lean or obese individuals. In obese adult SAT, long-chain PUFA (20:4, 20:5, and 22:6)-containing plasmenyl PC (PC P-) levels are increased, whereas in VAT, the level of 18 carbon acyl chain-containing PE P- is increased, showing adipose depot-specific differences in lipid composition^2^. Similarly, in the untargeted lipidomics results of 48 human SATs, PUFA-containing plasmalogen (PC P- and PE P-) showed a positive correlation with body mass index (BMI)^94^. In another study of obese-discordant twins, AA-containing PE P- was increased in the SAT of obese twins^86^. Overall, the studies suggest that plasmalogens in WAT are increased in obese conditions, which could be a compensatory adaptation to reduce increased oxidative stress caused by chronic inflammation^95^.

Metabolomics analysis of VAT from 53 adults revealed that plasmalogen levels are higher in pathogenic obese (BMI > 40 kg/m^2^ with metabolic syndromes) VAT than in healthy obese or healthy nonobese VAT^95^. On the other hand, in the study by Pietiläinen et al.^86^, the ratio of PUFA-containing PE P- (16:0/20:4 and 18:1/20:4) was significantly lower in pathogenic obese (BMI = 47.0–60.4 kg/m2) SAT and VAT than in obese or lean SAT and VAT in humans. In line with this finding, in a HF diet-induced obese mouse model, the content of PUFA-containing PE P- (16:0/20:4 and 18:1/20:4) is reduced in all three adipose depots, particularly in iWAT and gWAT^94^. The conflicting results shown in pathogenic obesity may be due to differences in criteria such as BMI and comorbidities. Sn-2 acyl chain hydrolysis of plasmalogen is mediated by calcium-independent, plasmalogen-selective, and tissue-specific phospholipase A2 enzymes (iPLA2)^96^, resulting in the production of lysoplasmalogen (LPC P- and LPE P-)^86^. iPLA2 levels have been reported to increase in obese conditions^97^, but LPE P- 18:0 is significantly decreased in the VAT of obese individuals compared to nonobese individuals^95^. In contrast, in pathogenic obesity, the content of LPE P- (16:0, 18:0, and 18:1) is increased compared to that in obese or nonobese individuals^95^. Untargeted phospholipid lipidomics performed by our group revealed that the level of LPE P-18:0 in human SAT has a negative correlation with BMI. We also observed a decrease in the LPE P- (16:0, 18:0, 18:1, 18:2, and 20:0) and LPC P-18:0 levels in iWAT of a HF diet-induced mouse model^94^. Additionally, significant decreases in LPE P- (16:0, 18:0, 18:1, 18:2, and 20:0) were observed in the gWAT of obese mice, and LPC P-18:0 and LPE P- (16:0, 18:1, 18:2, and 20:0) show significant decreases in the BAT of obese mice^94^. Pietiläinen et al.^86^ interpreted the decreased plasmalogen levels in pathogenic obesity compared to healthy obesity to be caused by the collapse of compensatory mechanisms against metabolic stress, indicating that plasmalogen homeostasis in adipose tissue could have a significant impact on obesity-related systemic metabolism.

Ceramides and their metabolites belonging to the sphingolipid family are major lipids associated with obesity-related pathologies^98–100^. Feeding mice an obesogenic HF diet results in an increase in Cer 16:0 levels in BAT and gWAT^100,101^, which is also observed in the VAT of obese humans^101^. Cer 16:0 modulates adipose function by compromising mitochondrial respiration in BAT and contributes to the development of obesity-associated insulin resistance^100–102^. In the gWAT of HF diet-induced obese mice, the levels of dihydroceramide (DHC) are increased^92^. DHC is an immediate precursor to ceramide, and the increased concentration of DHC in adipocytes inhibits adipogenesis and promotes cell death, which suppresses adipose tissue expansion in gWAT, thereby facilitating ectopic fat deposition in HF diet-induced obesity^103^. In addition, among various forms of ceramide, Lange et al.^2^ suggested that the accumulation of sphingadiene Cer (SPB 18:2; O2) in SAT and VAT is a hallmark of obesity, accounting for 19% of the ceramide subclass. However, the functional role of this lipid species in adipose tissue is still unknown and requires further investigation.

Blandin et al.^92^ examined the lipidome of extracellular vesicles (EVs) derived from gWAT and compared the differences in composition between lean and obese mice. Notably, the levels of ceramides in EVs derived from adipose tissue were lower in obese gWAT than in lean gWAT, while no depletion of Cer was found in EVs from isolated adipocytes of obese gWAT compared to lean gWAT^92^. These results suggest that changes in the EV lipidome are influenced not only by adipose cells but also by nonadipose cells. This finding highlights the complex interplay between various cell types in altering the EV lipidome.

## Lipidomics-based approach to identifying bioactive lipids that regulate adipocyte metabolism

**Fig. 1:**
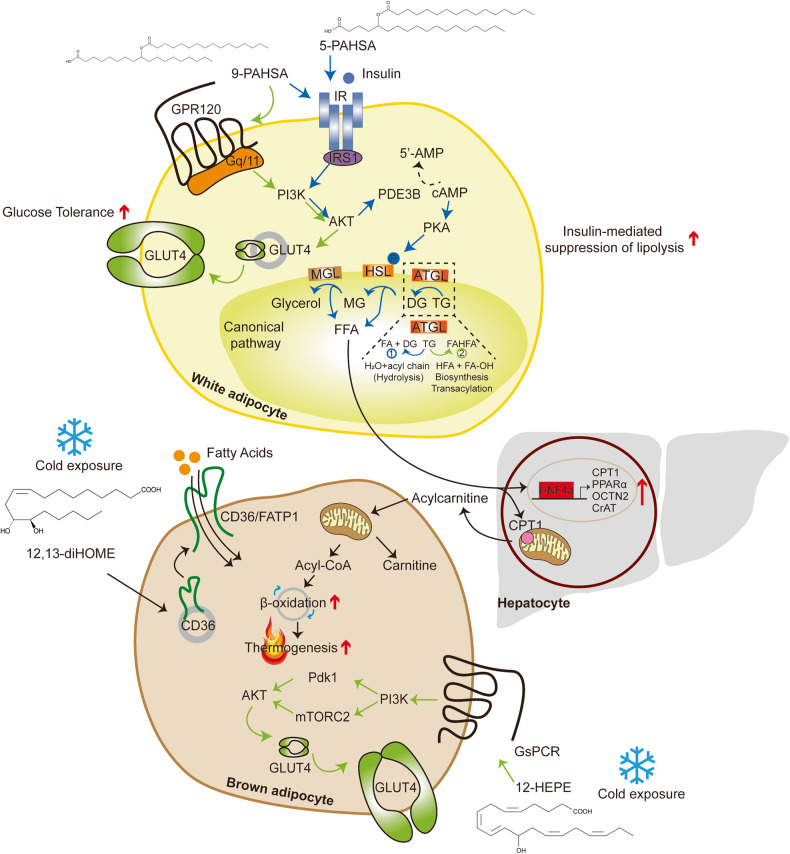
Bioactive lipid-mediated regulation of insulin downstream signaling and lipid metabolism in adipocytes: mechanistic overview. The schematic depicts the mechanisms of bioactive lipids identified by the lipidomics approach in adipocyte metabolism. The ester bond formation of FAHFAs is catalyzed by ATGL, and 9-PAHSA enhances insulin-stimulated glucose transport by enhancing GLUT4 translocation via the GPR120/PI3K/AKT axis. 5- and 9-PAHSA promote insulin-mediated suppression of lipolysis in white adipocytes. 12,13-DiHOME induces FA transporter (CD36) translocation, elevating FA uptake and producing substrates for thermogenesis. 12-HETE stimulates the GsPCR/PI3K/mTORC2/AKT pathway, enhancing GLUT4 translocation and contributing to improved insulin sensitivity. Circulating FAs activate HNF4α, promoting the gene expression of CPT1, PPARα, OCTN2, and CrAT, and they mediate hepatic production of acylcarnitine, which then serves as fuel for β-oxidation.

Despite various studies identifying lipid species involved in insulin sensitivity, mitochondrial metabolism, and thermogenesis of adipose tissue, this section mainly focuses on lipids that were recently identified by global lipidomics analysis of adipose tissue and serum. The mechanistic overview of these lipids is illustrated in Fig. 1.

### FAHFAs

A reduction in glucose transporter type 4 (GLUT4) expression in adipose tissue is one of the characteristics of obese or diabetic conditions in rodent models and human patients^104,105^, while GLUT4 overexpression reduces fasting hyperglycemia and improves glucose tolerance^106,107^. Based on the metabolically improved phenotype of GLUT4-overexpressing mouse models, Yore et al. hypothesized that GLUT4 overexpression may affect the lipidomes of adipose tissues by generating beneficial lipid mediators. The researchers performed a quantitative mass spectrometry analysis of iWAT obtained from mice overexpressing GLUT4 and identified a family of orphan lipids, namely, FAHFAs^54^.

Recently, Patel et al.^108^ demonstrated that adipose triglyceride lipase (ATGL) serves as a biosynthetic enzyme responsible for catalyzing the ester bond formation of FAHFAs in mammals, which was first suggested by Paluchova et al., revealing the release of FAHFAs from TG estolides during lipolysis via ATGL^109^. Yore et al. found the most abundant form of the family, 9-PAHSA, in BAT and WAT of wild-type (WT) mice as well as the SAT of humans, and the levels of 5- and 9-PAHSA in serum and WAT are positively correlated with insulin sensitivity in rodents and humans. 5-PAHSA improves glucose tolerance by enhancing insulin secretion and GLP-1 stimulation, while 9-PAHSA augments glucose uptake in response to insulin stimulation without altering the protein levels of GLUT1 or GLUT4 in adipocytes^54^. Interestingly, 9-PAHSA binds to and activates GPR120 in a dose-dependent manner, and abrogation of *Gpr120* attenuates the effects of PAHSAs on insulin-stimulated glucose transport mediated by GLUT4 translocation^54,110^. Moreover, 5- and 9-PAHSAs promote insulin-mediated suppression of lipolysis in WAT and insulin-mediated suppression of glucose production in the liver through the regulation of FFA levels in the bloodstream^111^, while 5-PAHSA-mediated de novo lipogenesis promotes energy consumption and lipid remodeling instead of lipid storage in gWAT^109^. In addition to insulin-sensitizing effects in adipocytes, 9-PAHSA blocks lipopolysaccharide-induced IL-12 secretion, and it reduces IL-1β and TNFα levels in bone-marrow-derived dendritic cells and the number of adipose tissue macrophages expressing IL-1β and TNFα in HF diet-fed mice, revealing its anti-inflammatory effects in adipose tissue^54^.

However, Pflimlin et al.^112^ challenged the beneficial effects of a combination of 5- and 9-PAHSA treatment. Despite the controversy over the effects of PAHSA, its potential therapeutic effects in various disease conditions are continuously being reported, including cognitive improvement in diabetic mice^113^, reduction in autoimmune type 1 diabetes incidence^114^, augmentation of insulin secretion via GPR40 in β-cells^115^, and alleviation of colitis in mice^116^. A comprehensive review of FAHFAs, from their structure to their involvement in physiology, has been reviewed elsewhere^117^.

### 12,13-DiHOME

Adipose tissue plays a crucial role in lipid metabolism by releasing lipid mediators that can influence systemic metabolism and taking up circulating bioactive lipids from the bloodstream as substrates or signaling molecules^60,118,119^. The unbiased and comprehensive analysis of bioactive lipid metabolites in serum using lipidomics approaches has offered a valuable source of information on potent lipid species.

To elucidate the link between thermogenic lipokines and BAT activation, Lynes et al.^118^ performed liquid chromatography-tandem mass spectrometry (LC‒MS/MS) using the plasma of human volunteers exposed to cold and discovered that 12,13-dihydroxy-9Z-octadecenoic acid (12,13-diHOME) levels are elevated in response to an acute cold challenge in humans and mice. 12,13-DiHOME, originally known to suppress neutrophil respiratory burst^120^, is synthesized from linoleic acid by cytochrome P450 and epoxide hydrolase and is secreted by BAT into the circulation under cold exposure and exercise^121^. The authors revealed a negative correlation between circulating 12,13-diHOME levels and BMI, insulin resistance, fasting plasma insulin, and glucose concentrations^118^, which is further supported by other large cross-sectional studies in humans^122^. Lyne et al.^118^ observed that cold challenge elevates 12,13-diHOME levels in adipose tissue by increasing lipolysis to provide substrates for soluble epoxide hydrolases and enhancing their gene expression, whereas a severe defect in classical BAT development prevents this increase. 12,13-DiHOME increases FA uptake in BAT by promoting the translocation of FA transporters^118^. This reduces circulating TG levels and enhances cold adaptation by augmenting lipid oxidation, especially the hydrolysis of TG, and providing fuel for thermogenesis. Chronic cold exposure also increases the production of soluble epoxide hydrolases, specifically in brown or beige adipocytes, leading to enhanced 12,13-diHOME biosynthesis. In line with this, exercise stimulated the secretion of 12,13-diHOME into the circulation in mice and humans, leading to greater skeletal muscle respiration^121^. Taken together, these findings indicate its pivotal role in thermogenesis and its potency to serve as a biomarker for BAT activation in humans^118^.

### 12-HEPE

Aiming to search for lipoxygenase (LOX) products that reflect the activity of this family of enzymes under cold conditions, Leiria et al.^119^ conducted LC‒MS/MS from the serum of mice that were housed in cold or thermoneutrality for 7 days. The levels of 12-LOX metabolites such as 12-hydroxyeicosapentaenoic acid (12-HEPE), 14-hydroxydocosahexaenoic acid (14-HDHA), and 12-hydroxyeicosatetraenoic acid (12-HETE) are elevated upon cold exposure; however, this increase is abolished in the absence of lipolysis in adipose tissue, emphasizing the pivotal role of the ATGL-dependent lipolytic pathway in the production of these metabolites^119^. Targeted lipidomics in BAT and iWAT of mice housed at cold or thermoneutrality validated that brown adipocytes are the cellular source of 12-LOX metabolite production in response to cold or β3-adrenergic stimulation in rodents and humans^119^. As a result, the absence of *Alox12*, which encodes 12-LOX, in UCP1^+^ adipocytes leads to impairment in cold adaptation and results in a reduction in whole-body oxygen consumption in response to norepinephrine stimulation. 12(*S*)-HEPE promotes glucose uptake by triggering G_s_PCR, which leads to PI3K-mTORC2-AKT activation and GLUT4 translocation to the plasma membrane and utilization in BAT, thereby improving glucose tolerance and insulin sensitivity in diet-induced obese (DIO) mice^119^. In healthy human subjects, β3-adrenergic stimulation leads to elevated levels of 12-HEPE and 14-HDHA in serum. Furthermore, there is a negative correlation between the plasma levels of 12-LOX products and BMI, insulin resistance, and leptin concentrations, while 12-LOX metabolites are positively correlated with BAT activity in humans^119^. Kulterer et al.^123^ observed higher levels of circulating 12-HEPE in cold-exposed human subjects with detectable BAT activity, suggesting that the potency of this metabolite in regulating glucose metabolism and thermogenesis is not limited to rodents but may also be relevant in humans.

### Acylcarnitine

Simcox et al.^60^ performed LC‒MS-based lipidomics analysis using the plasma of mice that were housed at either room temperature (RT) or cold conditions. Of the circulating lipids whose levels were increased by cold challenge, long-chain acylcarnitines were elevated, which was further validated by LC‒MS/MS^60^. As aging leads to the loss of BAT function and elevated susceptibility to hypothermia^124,125^, the authors compared plasma lipids between young and old mice housed at RT or cold conditions utilizing ultra-performance LC‒MS/MS (UPLC‒MS/MS)^60^. Regardless of the length of their chain, acylcarnitine levels are increased upon cold exposure, with this effect being particularly pronounced in young mice, although older mice exhibited higher basal concentrations of acylcarnitines at RT. Conversely, cold challenge elicits a decrease in plasma carnitine levels in both young and old mice^60^. To find the source of the circulating acylcarnitines, Simcox et al. analyzed the expression of genes involved in acylcarnitine metabolism in the liver, skeletal muscle, and BAT and observed that cold challenge elevates gene expression only in the liver concomitant with knockdown of *Cpt1a* and *1b* in the liver, thus reducing acylcarnitine levels in the bloodstream. Knockdown of *Cpt1a* and *1b* also reduces core body temperature, which is recovered by exogenous palmitoylcarnitine. Additionally, stimulation of nonshivering thermogenesis with the β3-adrenergic agonist CL316,243 elevates serum acylcarnitine levels^60^. Despite the lack of β3-adrenergic receptor expression in the liver, pharmacological stimulation still enhances hepatic gene expression involved in acylcarnitine metabolism, which is driven by FFAs released from WAT upon β3-adrenergic receptor agonism^60^. This response is further supported by the presence of HNF4α, which is a nuclear receptor activated by fatty acids and known regulators of *Cpt1* and *Cpt2* expression^126,127^. HNF4α regulates acylcarnitine metabolism and helps to maintain body temperature during cold challenge, and it augments the expression of HNF4α target genes upon palmitate treatment^60^. Uptake of circulating palmitoylcarnitine by brown adipocytes suggests a fuel source for thermogenesis in BAT, and promoting the production of acylcarnitine and exogenous palmitoylcarnitine improves thermoregulation and reverses cold sensitivity in old mice exposed to cold^60^. Although this study revealed that cold-induced liver-derived acylcarnitines provide a fuel source for BAT thermogenesis, further studies are required to understand the underlying molecular mechanism.

## Mechanisms and functions of lipid remodeling in adipose tissue

**Fig. 2:**
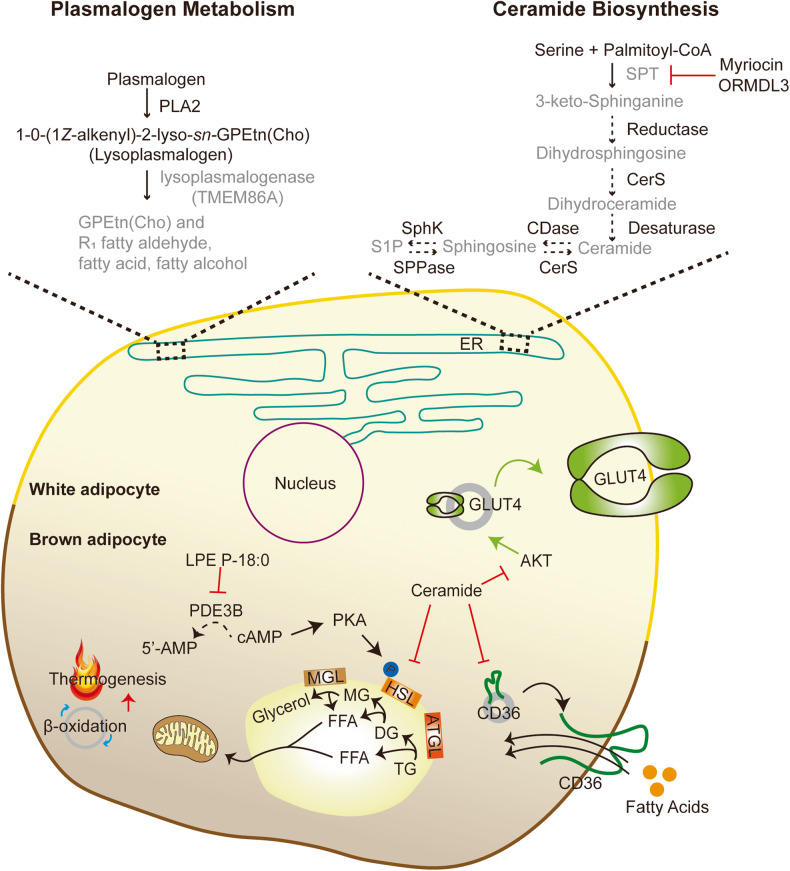
Effects of genetic deletion of lipid biosynthetic and metabolic enzymes on adipocyte metabolism. Enzymes and pathways involved in plasmalogen metabolism: TMEM86A has lysoplasmalogenase activity in adipocytes. Inhibiting the activity of TMEM86A leads to an increase in lysoplasmalogen and plasmalogen. LPE P-18:0, a lysoplasmalogen species, suppresses PDE3B activity, elevating intracellular cAMP levels. Increased levels of intracellular cAMP activate the PKA signaling pathway and induce lipolysis by phosphorylating HSL accompanied by enhanced thermogenesis. Enzymes and pathways involved in ceramide biosynthesis: Genetic abrogation of *Sptlc2* and *Ormdl3* regulates ceramide biosynthesis. Ceramides alleviate GLUT4 translocation via inhibition of AKT signaling, attenuate CD36 translocation, and suppress the activity of HSL, leading to impaired glucose tolerance, fatty acid uptake, and thermogenesis.

This section focuses on the enzymes responsible for lipid remodeling in adipocytes triggered by pathophysiological stimuli. We highlight the recent lipidomics studies conducted using transgenic mouse models that target lipid-modifying enzymes. Figure 2 provides a comprehensive summary the function of these enzymes in adipocyte metabolism.

### TMEM86A

Plasmalogens, a type of lipid found in adipocyte membrane phospholipids, are involved in the production of lipid mediators, and their dysregulation has been linked to obesity-related metabolic disease^86^. The biosynthesis of plasmalogens begins in the peroxisome and is completed in the endoplasmic reticulum. Plasmalogens can be hydrolyzed by PLA2 and further catabolized by phospholipase C, phospholipase D, and lysoplasmalogenase, and they may also be reacylated to reform plasmalogens^128^.

Our group recently demonstrated the lysoplasmalogenase activity of transmembrane protein 86A (TMEM86A) by LC‒MS-based global lipidomics analysis in adipose tissue of WT and adipocyte-specific *Tmem86a* KO mice. Obesity is associated with reduced lysoplasmalogen levels and increased TMEM86A protein levels in adipose tissue of rodent models and human patients. TMEM86A deletion elevates lysoplasmalogen and plasmalogen levels in fat pads, particularly LPC-P 18:0 and LPE P-18:0, compared to those in WT mice. In vitro and in vivo LPC P-18:0 and LPE P-18:0 supplementation stimulate intracellular cAMP-dependent protein kinase A (PKA) signaling by inhibiting phosphodiesterase 3B (PDE3B) activity, which then facilitates mitochondrial oxidative metabolism and protects against diet-induced obesity^94^. Although plasmalogen was previously reported to be abundantly found in brain and heart tissues, Lange et al.^2^ recently demonstrated that plasmalogen is also enriched in human WAT with depot-specific signatures. Shark liver oil supplementation containing a rich source of alkylglycerol in overweight or obese subjects leads to lipidome changes in plasma and circulatory white blood cells, notably plasmalogen levels, and this results in reduced total free cholesterol, TGs, and C-reactive protein levels in plasma^129^. In line with this finding, circulating plasmalogen levels are negatively correlated with hypertension, prediabetes, T2D, cardiovascular diseases, and obesity^96^, suggesting that the regulation of plasmalogen homeostasis in humans may play a vital role in lipidome remodeling in humans, providing therapeutic approaches against metabolic diseases.

### PSD

PE is particularly enriched in cristae, supporting the function of the complexes involved in the electron transport chain^130^; therefore, impaired mitochondrial PE is known to cause severe mitochondrial disease, cristae malformation, and abrogated oxidative phosphorylation^131,132^. As no known importer for PE exists^133^, phosphatidylserine decarboxylase (PSD) is likely to exclusively generate mitochondrial PE.

Johnson et al.^70^ performed lipid profiling of mitochondria isolated from BAT to investigate altered mitochondrial lipid compositions under interventions such as ambient temperature. Among various phospholipids, PE, which is reported to be elevated in skeletal muscle by exercise^70^, is significantly increased upon cold exposure and is the only lipid class that was decreased by thermoneutrality. In addition, phospholipidome analysis of BAT mitochondria from *Ucp1-*deficient mice resembles the lipidome alterations observed in thermoneutrality, and the authors elucidated that PE is the only energy-responsive lipid among the three models^70^. To further investigate the role of mitochondrial PE in BAT thermogenesis, Johnson et al. generated UCP1-expressing cell type-specific PSD KO mice to inhibit mitochondrial PE synthesis. The KO mice exhibit larger lipid droplets and fibrosis in BAT accompanied by a considerable reduction in cold tolerance, indicating impaired thermogenic capacity^70^. Furthermore, the absence of PSD in UCP1+ cells resulted in reduced mitochondrial density, cristae malformation, decreased lipolysis, and upregulated DGAT2 expression^70^. In particular, elevated DGAT2 expression is linked to the recruitment of lipid droplets to mitochondria, forming peridroplet mitochondria^134,135^. Although malformed cristae do not alter UCP1 protein abundance in mitochondria, they blunt UCP1-dependent respiration, suggesting lowered oxidative capacity per unit of mitochondria and emphasizing the importance of mitochondrial PE for UCP1 function^70^.

### ORMDL3

Sphingolipids are essential components of the cell membrane in eukaryotic cells and serve as signaling molecules^136–139^. SPT comprises three subunits, namely, SPTLC1, SPTLC2, and SPTLC3, and is the first rate-limiting step in de novo ceramide biosynthesis^140^. The buildup of sphingolipids in various tissues disrupts glucose and lipid metabolism in the obese state^136–139^.

Orosomucoid-like (ORMDL) proteins are known to negatively regulate the activity of SPT,^141^ and ORMDL sphingolipid biosynthesis regulator 3 (ORMDL3) is a member of the ORMDL gene family. Notably, *Ormdl3* has been identified as a gene associated with obesity via an integrative genomic approach, and its expression shows a negative correlation with BMI^142^.

Song et al.^143^ observed that *Ormdl3* exhibits the highest expression in BAT and is upregulated by cold exposure in adipose tissue concomitant with reduced expression in obese mice and humans, suggesting its potential role in thermogenesis. Mice with *Ormdl3* deletion fail to maintain core body temperature during cold exposure and exhibit reduced thermogenic gene expression along with attenuated stimulation of β3-adrenergic receptors in BAT and iWAT. HF diet feeding leads to more body weight gain in *Ormdl3* KO mice accompanied by increased adiposity and insulin resistance, worsening the overall metabolic phenotypes associated with diet-induced obesity^143^. To examine whether lipid remodeling contributes to the aforementioned phenotypes observed in *Ormdl3* KO mice, Song et al. performed nontargeted lipidomics analysis in WAT from WT and *Ormdl3-*deficient mice. The authors observed elevated ceramide levels in WAT from KO mice^143^, consistent with previous studies^144,145^. Pharmacological inhibition of de novo ceramide synthesis recovers impaired thermogenesis in *Ormdl3* KO mice^143^.

### SPT

To examine whether the sphingolipid compositions in adipose tissue differ in diabetic or nondiabetic obese subjects, Chaurasia et al.^69^ analyzed the lipidome composition of SAT and reported elevations in various sphingolipid levels. Additionally, Turpin et al. sought to identify the altered acyl-chain ceramide profiles in WAT of obese human subjects and found that CerS6 expression and its main product, Cer 16:0, positively correlate with obesity and insulin resistance^101^. A more recent study confirmed that Cer 16:0 with the usual sphingoid base sphingosine (d18:1) is the most abundant ceramide in human WAT, indicating its vital role in adipose tissue metabolism and suggesting the association between ceramide profiles and the development of obesity in humans^2,101^. In support of the data from human studies, pharmacological inhibition of sphingolipid biosynthesis with myriocin in DIO mice leads to reduced fat mass and adiposity along with improved insulin sensitivity and enhanced energy expenditure primarily due to the stimulation of thermogenesis in iWAT^69^. Adipocyte-specific *Sptlc2*-deficient mice were generated to address adipose sphingolipids contributing to these phenotypic changes^69^. As expected, the deletion of *Sptlc2* reduced ceramide and dihydroceramide levels in primary adipocytes and adipose depots. In line with the pharmacological inhibition, genetic disruption of *Sptlc2* in DIO mice displays similar phenotypes, such as reduced fat mass, improved energy expenditure, and thermoregulation^69^. Both pharmacological inhibition of sphingolipid synthesis and genetic deletion of *Sptlc2* prevent the differentiation of adipocytes^69,146^, contributing to reduced fat mass. Chaurasia et al.^69^ also observed elevated thermogenic gene expression and mitochondrial activation in the iWAT and BAT of adipose-specific *Sptlc2*-depleted mice, further supporting that adipose sphingolipids, not macrophage sphingolipids, function as intrinsic mediators in adipose tissue metabolism. In addition to its improved whole-body bioenergetics, the regulation of sphingolipid production exhibits markedly improved whole-body insulin action^69^. Conversely, adipose sphingolipid profiling upon cold challenge or β3-adrenergic agonism reveals a reduction in intermediates involved in sphingolipid production, such as ceramide, dihydroceramide, and sphinganine, in WAT, indicating that regulation of sphingolipids may contribute to the thermogenic properties of WAT. Cer 2:0, which elevates endogenous sphingolipid levels through the salvage pathway, and D609, a sphingomyelin synthase inhibitor, attenuate mitochondrial respiration in iWAT primary adipocytes^69^ and suppress the expression of thermogenic genes in beige adipocytes^147^. Consistent with rodent studies, concomitant treatment of Cer 2:0 with a β-adrenergic agonist in mature adipocytes differentiated from human SAT precursors inhibits the induction of thermogenic genes, implying that the effects of lipid remodeling in adipocyte metabolism are not only limited to rodents but can also be applied to humans to assist combating metabolic diseases.

As a follow-up study, Chaurasia et al.^100^ performed mass spectrometry, quantifying 38 sphingolipids in BAT from mice fed a normal chow diet or HF diet, and found that Cer 16:0 levels are elevated upon HF diet feeding and that inhibition of Cer 16:0 synthesis in the liver led to improved glucose tolerance and insulin sensitivity along with reduced body fat in *ob/ob* mice^148^. β-Adrenergic agonism and a HF diet reciprocally regulate enzymes involved in sphingolipid metabolism^100,146^. Chaurasia et al.^100^ revealed reduced circulating ceramides in UCP1-expressing cell type-specific *Sptlc2*-deficient mice, indicating that UCP1^+^ adipocytes release ceramides into the bloodstream. In addition, *Sptlc2* deletion in UCP1-expressing cell types reduces fat mass and enhances energy expenditure along with improving insulin tolerance, suggesting that ceramides in thermogenic adipocytes contribute to impaired thermogenesis and the development of obesity. On the other hand, acid ceramidase accumulates ceramides and other sphingolipids by slowing the degradation of ceramide, and UCP1-expressing cell type-specific ablation of *Asah1* leads to increased HF diet-induced adiposity as a result of decreased energy expenditure and insulin intolerance^100^. Inhibition of ceramide synthesis and the accumulation of ceramides reciprocally regulate thermogenic genes, thermoregulation, and mitochondrial activity in UCP1^+^ adipocytes, further supporting that the regulation of ceramide synthesis in UCP1^+^ adipocytes contributes to their thermogenic capacity and energy expenditure. Mechanistically, ceramides suppress the activity of enzymes such as AKT and hormone-sensitive lipase, which augment the translocation of glucose transporters and lipolysis, respectively, and modulate fatty acid uptake rates^100^.

Taken together, numerous data suggest that inhibiting the synthesis of sphingolipids may attenuate disrupted metabolic phenotypes caused by the accumulation of sphingolipids in adipose tissue^69,100,102,143^. However, adipocyte-specific deletion of *Sptlc1* leads to loss of adipose tissue mass in an age-dependent manner along with adipocyte death and metabolic dysfunction^149^, and adipocyte-specific *Sptlc2* deficiency contributes to metabolic dysfunction in other tissues, such as the liver, causing hepatosteatosis and insulin resistance^146^, which may explain the unexpected increase in *Sptlc2* transcripts in the liver of adipose-specific *Sptlc2* KO mice^69^. Therefore, completely inhibiting the production of sphingolipids and ceramides across the entire system carries a significant risk of adverse effects^150^. Xu et al.^59^ showed that cold exposure elevates sphingomyelin and ceramide in iWAT, accompanied by enhanced sphingolipid biogenesis genes and reduced sphingolipid breakdown-related genes, which is contradictory to a recent report^100^. The increased availability of FFAs induced by cold exposure may partially contribute to the elevated sphingolipid levels observed in the iWAT of mice, as FA serves as a substrate for sphingolipid biosynthesis. Lee et al.^146^ reported that the modified expression of genes involved in ceramide biosynthesis is shifted toward the synthesis of sphingosine-1-phosphate (S1P) via ceramide in obese conditions and that adipose-specific *Sptlc2* deficiency reduces S1P and S1P receptor 1 (S1PR1) levels. As S1P induces the proliferation of preadipocytes and adipogenesis via the activation of PPARγ and SREBP-1c, reduced S1P levels contribute to impaired adipogenesis and the development of adipose tissue, causing insulin resistance^146^.

## Discussion

Adipose tissue lipid remodeling is a complex process that plays a critical role in the regulation of metabolic homeostasis. Dysregulation of this process can lead to the development of metabolic disorders, highlighting the importance of understanding the molecular mechanisms that govern lipid metabolism in adipose tissue. Recent advances in lipidomics and metabolomics have provided a more comprehensive view of the lipid composition of adipose tissue and its role in metabolic health, opening up new avenues for therapeutic interventions. Furthermore, emerging evidence suggests that targeting adipose tissue lipid remodeling may represent a promising strategy for improving metabolic health and preventing the onset of metabolic disorders.

While we have not discussed it in detail in this review, it is worth noting that metabolite tracing technology is a powerful tool that can be used in conjunction with lipidomics studies to gain insights into the metabolic pathways involved in lipid metabolism. Recently, Wunderling et al.^151^ developed a tracing technology based on alkyne-labeled fatty acids that can track the metabolism of multiple fatty acids simultaneously and quantitatively, allowing direct investigation of TG cycling with molecular species resolution.

In this review, we have summarized recent lipidomics analyses of rodent models and highlighted several findings that have relevance to clinical studies, and vice versa. However, we acknowledge the limitations of extrapolating findings from rodent studies to humans, and we emphasize the importance of validating results in human studies to ensure their clinical relevance. For example, studies by Spalding et al.^152^ have shown that lipid turnover is slower in humans than in rodents, suggesting that the dynamic regulation of lipid remodeling may have species-specific characteristics. Therefore, it is crucial to consider multiple factors when interpreting the clinical relevance of findings from rodent studies.

In summary, recent lipidomics-based approaches have improved our understanding of the molecular mechanisms underlying adipose tissue lipid remodeling and have identified key enzymes and signaling pathways that regulate lipid metabolism. Future research on innovative techniques for modulating key molecular targets to rescue healthy adipose lipidomes could advance our understanding of the interplay between adipose-derived lipids and metabolic disorders and lead to more effective treatments for these diseases.
